# Supplementary material for: Chronic Disruption of the Late Cholesterol Synthesis Leads to Female-Prevalent Liver Cancer
Source: Cancers (Basel). 2020 Nov 9;12(11):3302. doi: 10.3390/cancers12113302 (PMC7695248; doi:10.3390/cancers12113302)
Supplement: Supplementary file 1 [file cancers-12-03302-s001.zip › cancers-949182-proof-resubmit.pdf]

cancers

SUPPLEMENTARY FIGURES.

Figure S1. Patho-histological features of *Cyp51* KO mice.
Figure S2. The KO mice liver and body weights at different ages, both sexes.
Figure S3. Immunohistochemical expression of *Tgfb1* and *Ctnnb1*.
Figure S4. qPCR expression profiles of TGF-β and Wnt signalling markers.
Figure S5. TF networks of 24M KO mice.

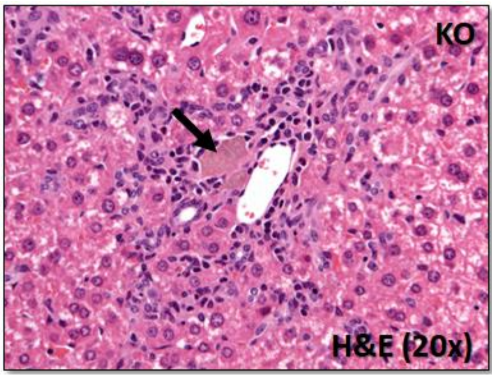

Figure S1. Patho-histological features of *Cyp51* KO mice. In livers of aging mice, yellow pigment likely representing lipofuscin pigment was observed in areas of pronounced ductular reaction, sometimes surrounded by other hepatic cells, evaluated as macrophages. Accumulation of yellow pigment in the liver of *Cyp51* KO mice (arrow)., (H&E, original magnification x200); N=6-10 mice/group. KO, *Cyp51* KO.

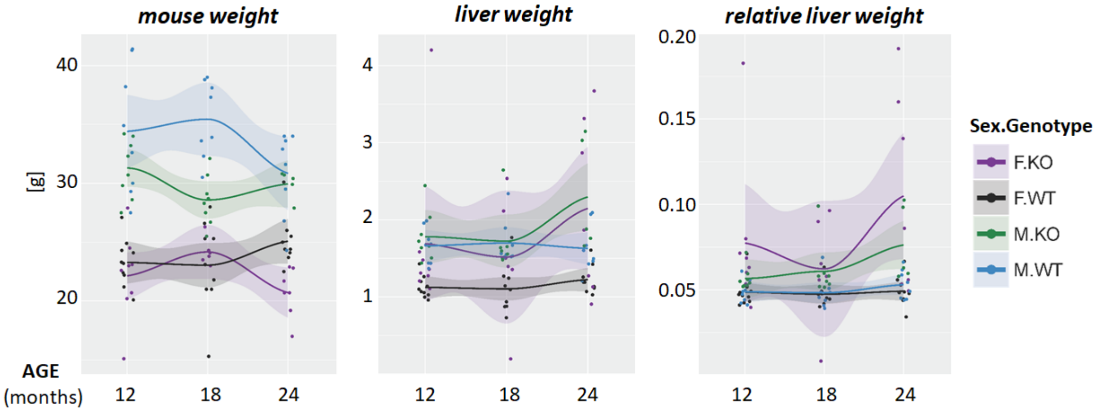

Figure S2. Comparison of mouse liver/body/relative weights: relative weights were significantly elevated in 24M KO females, who had the highest incidence of liver tumors. The KO mice of both sexes have significantly increased liver and decreased body weight at different ages. 5-10 mice were analysed in each group. G, gram; KO, *Cyp51* KO; WT, wild-type; F = female; M = male.

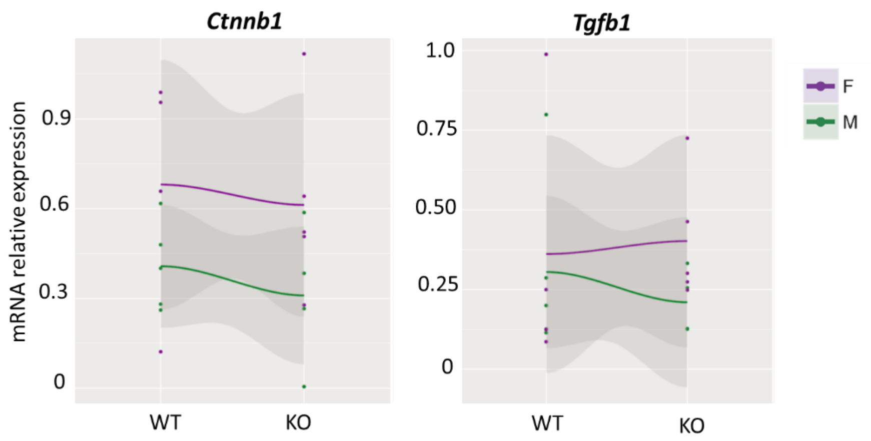

Parameter	Group 1	Group 2	Mean diff.	Pr(>F)
Ctnnb1	F	M	0.29	0.051 .
Tgfb1	F	M	0.07	0.343

Figure S3. qPCR expression profiles of TGF-β1 and Wnt signalling markers. Genotype profiles of qPCR expression measurements with statistically differences of selected genes *Ctnnb1* and *Tgfb1* in 24M KO mice. Light color bands represent 95% confidence interval and dots represent individual measurements. (N= 3-4 mice per sex/genotype group); KO, *Cyp51* KO; WT, wild-type, ~~β –cat~~*Ctnnb1*, β-catenin; F, female; M, male; p<0.1, * p<0.05, ** p<0.01, *** p<0.001.

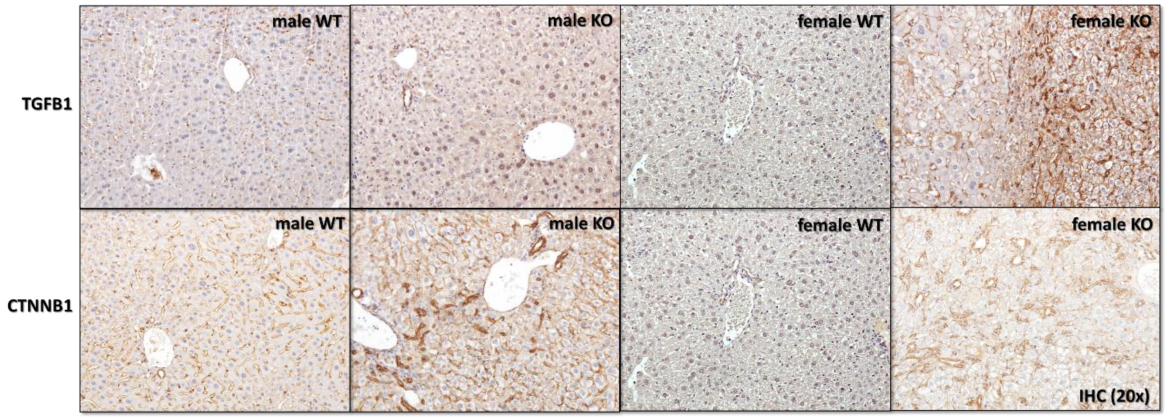

Figure S4. Immunohistochemical expression of TGF-β1 and β-catenin as potential markers of hepatocarcinogenesis in 24M.KO mice. A similar observation was made in 12M.KO mice (images not included). Original magnification, x200. TGFB1; tumor growth factor 1; CTNNB1, β-catenin; KO, *Cyp51* KO; WT, wild-type.

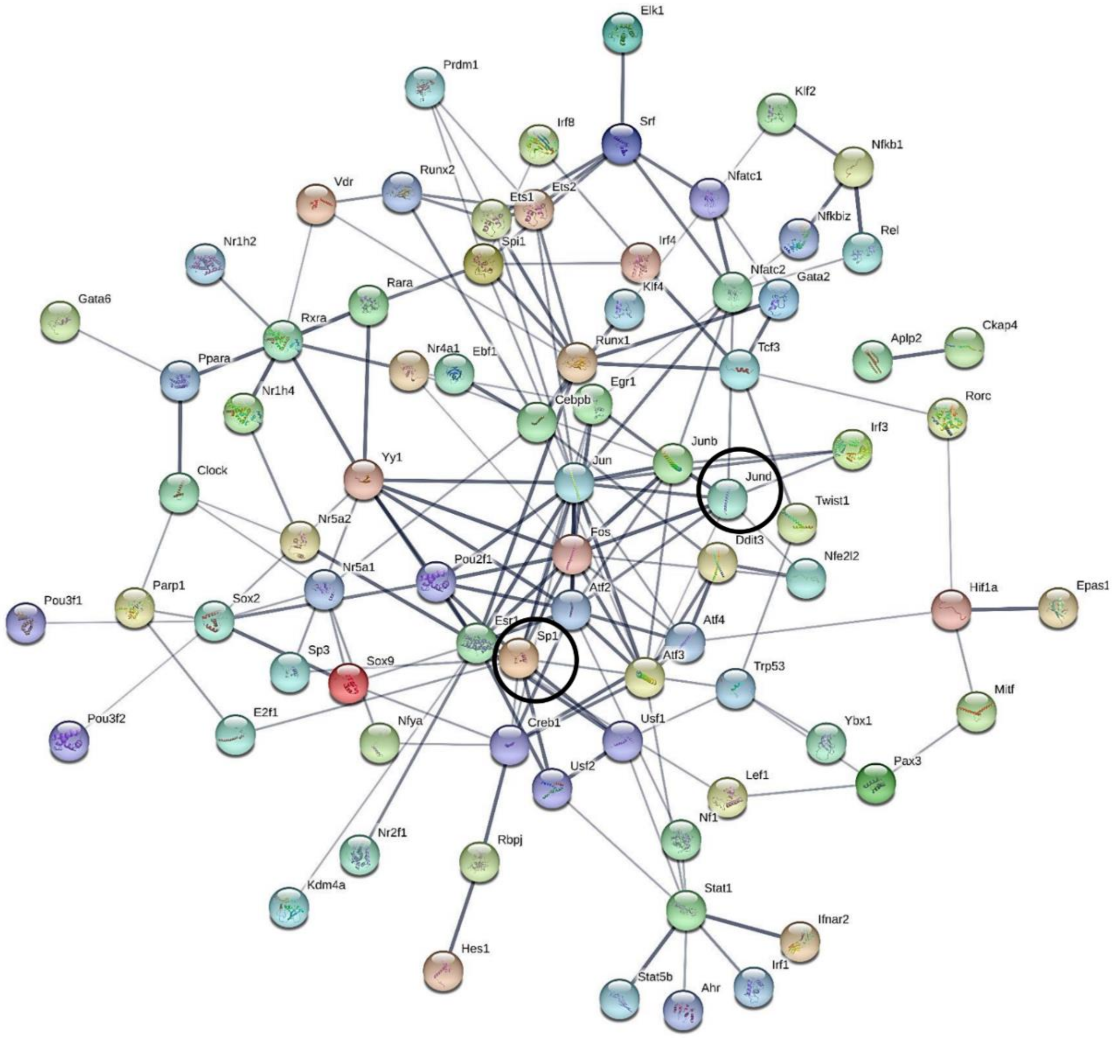

Figure S5. TF networks of 24M KO mice. TF networks generated with STRING show known and predicted protein-protein interactions. Network nodes represent TFs, produced by a single, protein-coding gene locus. For TFs with the known or predicted 3D structure, the structure is represented within nodes; empty nodes represent TFs of unknown 3D structure. Edges indicate protein-protein associations, i.e. proteins jointly contributing to a shared function and not necessarily physically binding each other. Nodules representing TFs SP1 and JUND are surrounded by black lines. KO, *Cyp51* KO.

© 2020 by the authors. Submitted for possible open access publication under the terms and conditions of the Creative Commons Attribution (CC BY) license (http://creativecommons.org/licenses/by/4.0/).
